# A Warning

**Published:** 1911-06-10

**Authors:** E. W. Morris

**Affiliations:** Secretary. London Hospital, Whitechapel, E.


### INSTITUTIONAL LETTER-BOX.

### A WARNING.

Sir,—May I be allowed to warn matrons and others of a woman named Catherine Dunlop who, when applying for places, says that she was trained at the London Hospital, which is quite untrue. She of course cannot produce any certificate, which should be asked for before anyone engages a London Hospital trained nurse. A careful register is kept at the London of all nurses who have gone through our training.—Yours faithfully,

London Hospital, E. W. Morris, Secretary.

Whitechapel, E., June 3.

